# Special Issue “State-of-the-Art Cancer Immunotherapies—2nd Edition”

**DOI:** 10.3390/ijms27093740

**Published:** 2026-04-23

**Authors:** Hisashi Nagase, Takuma Kato, Takayuki Yoshimoto

**Affiliations:** 1 Department of Parasitology, Shinshu University School of Medicine, 3-1-1 Asahi, Matsumoto 390-8621,Japan; 2 Division of Cancer Immune Multicellular System Regulation, Center for Cancer Immunotherapy and Immunobiology, Graduate School of Medicine, Kyoto University, CCII Bristol Myers Squibb Building, Yoshida-Konoe-cho, Sakyo-ku, Kyoto 606-8501, Japan; 3 Department of Immunoregulation, Institute of Medical Science, Tokyo Medical University, 6-1-1 Shinjuku, Shinjuku-ku, Tokyo 160-8402, Japan

Cancer immunotherapy harnesses the immune system to combat tumor cells. In recent years, significant advances have been achieved, particularly with adoptive cell transfer using chimeric antigen receptor (CAR) T-cells [1,2] and immune checkpoint inhibitors (ICIs), such as antibodies targeting programmed cell death 1 (PD-1) [3]. CAR molecules are composed of an extracellular antigen-recognition domain and intracellular signaling domains, including CD3ζ and one or more co-stimulatory signaling domains (CSSDs). Second- and third-generation CAR T-cells incorporate one or two CSSDs, respectively, which significantly influence T-cell activation, persistence, and overall function. Despite these advances, relapse remains a major challenge even in well-established therapies such as CD19-targeted CAR T-cells. This is largely attributed to limited in vivo persistence of CAR T-cells and antigen loss on tumor cells. Additionally, CAR T therapies have shown limited efficacy against solid tumors. One of the key barriers to long-term efficacy is T-cell exhaustion, a dysfunctional state characterized by reduced proliferation, impaired cytotoxicity, and increased expression of inhibitory receptors such as PD-1. CAR T-cells are particularly susceptible to exhaustion due to both antigen-dependent stimulation and antigen-independent “tonic signaling” mediated by the CAR itself during manufacturing [4]. This dual mechanism makes exhaustion in CAR T-cells more complex than in conventional T-cells and difficult to fully control. This editorial provides a brief overview of recent advances in cancer immunotherapy, focusing on strategies to overcome these limitations of CAR-T cell therapy [5,6] and its new target molecules [7,8], as well as the benefits and potential tumorigenic risks associated with stem cell immortalization [9].

CAR T-cell therapy has demonstrated remarkable clinical efficacy, particularly in hematologic malignancies such as B-cell cancers, and is now approved and widely used for relapsed or refractory cases. Yet, its broader clinical impact is limited by biological, technical, and economic challenges. The tumor microenvironment (TME) represents a major barrier in solid tumors. It is characterized by hypoxia, nutrient deprivation, and a dense extracellular matrix, all of which impair T-cell infiltration and function. Additionally, immunosuppressive cells such as tumor-associated macrophages inhibit CAR T-cell activity through cytokines and immune checkpoint interactions like PD-L1. Tumor heterogeneity further complicates treatment, as variable antigen expression allows some cancer cells to evade targeting. Antigen loss through mechanisms such as downregulation or trogocytosis also contributes to resistance and relapse. To address these issues, advanced genetic engineering strategies have been developed [5]. Modifying CAR constructs, particularly through humanization of domains, reduces immunogenicity and enhances persistence. T- cell exhaustion is being tackled through gene-editing approaches. Overexpression of transcription factors such as FOXO1 [10,11] and c-Jun [12], which maintain a stem-like phenotype, along with epigenetic regulators like SUV39H1 [13], enhances durability and anti-tumor activity.

Innovations in CAR design further enhance functionality [5]. Metabolic armoring with interleukin-10 (IL-10) restores mitochondrial function and reverses exhaustion [14], while dynamic CAR expression using CTLA-4 cytoplasmic domains reduces continuous activation and mitigates antigen loss caused by trogocytosis [15]. Additionally, incorporation of constitutively active c-Kit signaling (KITv) provides enhanced co-stimulatory signaling, improving resistance to immunosuppressive cytokines such as TGF-β, though safety concerns remain [16]. Targeting strategies for solid tumors are also evolving. Combinatorial CAR designs, including dual, bicistronic, and tandem CARs, enable recognition of multiple antigens, reducing escape. Novel targets such as B7-H3 [17] and nfP2X7 [18] offer broader tumor specificity, while tumor-agnostic approaches like amphiphile tagging allow CAR T-cells to recognize artificially introduced antigens, bypassing natural antigen limitations [19].

The choice of CSSD plays a crucial role in modulating CAR T-cell behavior. CAR T-cells incorporating a 4-1BB-derived CSSD are more resistant to tonic signaling and exhibit greater persistence and sustained effector function compared to those with CD28-derived CSSD, which tend to undergo earlier exhaustion [20]. Recent studies have explored alternative CSSDs, including those derived from herpes virus entry mediator (HVEM), a member of the TNF receptor superfamily [6]. Using HIV Env-targeting CAR T-cells as a model, comparative analyses of CD28-, 4-1BB-, and HVEM-based CAR constructs revealed that HVEM-CAR T-cells exhibited the lowest levels of exhaustion and the highest proliferative capacity upon antigen stimulation. These cells were also less susceptible to PD-L1-mediated suppression and maintained strong cytotoxic activity against target cells. Mechanistically, although CD28-CAR T-cells exhibited strong tonic signaling and pronounced CAR clustering, contributing to early exhaustion, HVEM-CAR T-cells showed reduced CAR clustering and weaker CD3ζ phosphorylation, indicating lower tonic signaling. These findings suggest that HVEM-derived CSSDs confer resistance to CAR T-cell exhaustion by minimizing tonic signaling and preserving functional activity. This highlights the importance of optimizing CAR design, particularly the choice of co-stimulatory domains, to improve persistence, reduce exhaustion, and enhance therapeutic efficacy in future CAR T-cell therapies.

Currently approved CAR T-cell products are autologous, meaning each therapy is derived from an individual patient’s cells. While this personalized approach ensures compatibility, it introduces substantial variability between batches, complicates manufacturing, and increases costs, thereby restricting widespread accessibility. Manufacturing remains a critical bottleneck. CAR T-cell production is complex, time-intensive (typically 3–4 weeks), and costly due to stringent good manufacturing practice (GMP) requirements, specialized facilities, and logistics [5]. These challenges limit patient access and delay treatment. Emerging solutions include decentralized and point-of-care manufacturing, as well as allogeneic “off-the-shelf” products derived from healthy donors, which may improve scalability and consistency. Technological advances aim to streamline production. Non-viral gene delivery methods such as the Sleeping Beauty transposon system offer safer and more cost-effective alternatives to viral vectors. Hybrid systems combining adeno-associated virus (AAV) and transposons improve integration efficiency and safety. Lipid nanoparticle-based approaches significantly reduce production time to as little as 1–3 days. Automation and closed-system manufacturing platforms are also gaining attention. These systems reduce contamination risk, improve reproducibility, and lower operational costs, although they currently lack flexibility and require high initial investment. Innovations such as microfluidic bioreactors and AI-driven process control promise further improvements in efficiency and scalability.

In addition to efficacy, treatment-related toxicities remain a critical consideration in CAR T-cell therapy. Among these, cytokine release syndrome (CRS) is the most common acute adverse event, characterized by systemic inflammation driven by elevated cytokine levels, while immune effector cell-associated neurotoxicity syndrome (ICANS) represents a distinct and potentially severe neurologic complication [21,22]. Other clinically relevant toxicities include prolonged cytopenias, infections, and B-cell aplasia resulting from on-target and off-tumor effects [23]. Although current management strategies, such as the use of IL-6 receptor blockade and corticosteroids, have improved the safety profile of CAR T-cell therapy, these adverse effects remain a major challenge in the broader clinical application of CAR T-cell therapy.

Thus, overcoming the limitations of CAR T-cell therapy will require integrating advances in genetic engineering, targeting strategies, and manufacturing technologies. The development of safer, more durable, cost-effective, and scalable solutions, including allogeneic platforms, has the potential to increase access to CAR T-cell therapy and establish it as a mainstream cancer treatment.

As promising new target molecules for cancer immunotherapy, nuclear receptor subfamily 4 group A member 1 (NR4A1), a member of the NR4A orphan nuclear receptor family [7], and glycogen synthase kinase-3 (GSK-3), particularly the GSK-3β isoform [8], have recently emerged.

The tumor microenvironment (TME) is a complex network of tumor cells and surrounding host components, including stromal and immune cells, that collectively regulate tumor progression. Among these regulators, NR4A1 has emerged as a key transcription factor controlling both metabolic and immune processes [24]. It modulates glycolysis, lipid synthesis, and amino acid metabolism, thereby supporting tumor growth. In addition, NR4A1 shapes immune responses in a context-dependent manner: it suppresses T-cell proliferation and cytokine production, regulates macrophage differentiation, inhibits dendritic cell activation, reduces NK cell cytotoxicity, and restricts B-cell expansion, ultimately influencing tumor progression and immune evasion. Given these diverse roles, NR4A1 represents a promising therapeutic target. Strategies including small-molecule inhibitors and proteolysis targeting chimera (PROTAC)-mediated degradation have shown potential to enhance anti-tumor immunity, for example, by increasing tumor-infiltrating B-cells and suppressing tumor growth [7]. However, therapeutic efficacy is highly dependent on the cellular composition of the TME and the context-specific functions of NR4A1. Notably, NR4A1 also exerts protective effects in certain immune subsets, such as patrolling monocytes that eliminate metastatic cells. Therefore, careful patient selection and a deeper understanding of NR4A1’s cell-specific functions are essential for safely and effectively targeting this pathway in cancer immunotherapy.

Dendritic cells (DCs) are professional antigen-presenting cells that bridge innate and adaptive immunity by activating T-cells through antigen presentation, including cross-presentation to CD8^+^ T-cells [25]. This function is critical for anti-tumor and anti-viral immunity. However, DC-based cancer vaccines have shown limited efficacy due to tumor-induced dysfunction. GSK-3β is a key regulator of DC function, influencing maturation, cytokine production, antigen presentation, and survival [26]. Although its inhibition can enhance DC activation and T-cell responses, results remain inconsistent across studies. To address this, a study using a DC-specific genetic deletion model of GSK-3β revealed a dual role for GSK-3β that is independent of β-catenin [8]. Loss of GSK-3β enhanced early CD8^+^ T-cell activation through increased cross-priming, but impaired the formation and maintenance of memory T-cells. Notably, GSK-3β-deficient DCs did not show increased β-catenin activity, and gene expression analyses confirmed distinct transcriptional programs, indicating that GSK-3β regulates DC function independently of canonical Wnt signaling. Mechanistically, GSK-3β modulates pathways such as IL-2/STAT5, TNF-α/NF-κB, and IFN-γ signaling, which are critical for balancing effector and memory T-cell responses. These findings suggest that temporal regulation of GSK-3β is crucial. Transient inhibition may enhance early anti-tumor immunity, whereas sustained inhibition could impair long-term immune memory. Overall, GSK-3β represents a promising but complex therapeutic target, and precise, time-dependent modulation may improve the efficacy of DC-based cancer immunotherapy.

Regenerative medicine, which utilizes stem cells with self-renewal and differentiation capacities to repair damaged tissues, is currently attracting considerable attention [27]. Among them, mesenchymal stromal/stem cells (MSCs) are widely used due to their multipotency, low tumorigenic risk, and strong immunomodulatory effects. Their therapeutic benefits are largely mediated by paracrine mechanisms, including the secretion of cytokines, growth factors, and exosomes, which has led to increasing interest in cell-free therapies based on MSC-derived secretomes [28,29,30]. However, primary MSCs are limited by replicative senescence caused by telomere shortening. To overcome this limitation, immortalized MSCs have been developed through genetic modification [9]. Introduction of hTERT extends cellular lifespan, while additional factors such as BMI1 or c-MYC can further enhance proliferation. Viral oncogenes (e.g., SV40, HPV E6/E7) are also effective but pose higher risks of genomic instability. Recent technologies, including CRISPR/Cas9 and inducible immortalization systems, offer more controlled and potentially safer approaches. Safety switches such as inducible caspase-9 further improve risk management. Immortalized MSCs enable large-scale, consistent production and show therapeutic potential in various disease models. Nevertheless, concerns regarding tumorigenicity, genetic instability, and immunogenicity remain. Cell-free therapies using immortalized MSC-derived secretomes may reduce these risks, although variability and potential oncogenic signaling require attention. Thus, while immortalized MSCs represent a promising platform, ensuring safety and regulatory compliance is essential for their clinical application.

## Data Availability

No new data were created or analyzed in this study. Data sharing is not applicable to this article.
